# Retention in care and adherence to HIV and AIDS treatment in Anambra State Nigeria

**DOI:** 10.1186/s12879-019-4293-8

**Published:** 2019-07-22

**Authors:** Chukwuma David Umeokonkwo, Chima Ariel Onoka, Pearl Adaoha Agu, Edmund Ndudi Ossai, Muhammad Shakir Balogun, Lawrence Ulu Ogbonnaya

**Affiliations:** 1 Department of Community Medicine, Federal Teaching Hospital Abakaliki, Ebonyi State, Nigeria; 2 Nigeria Field Epidemiology and Laboratory Training Programme, Abuja, Nigeria; 3 Department of Community Medicine, University of Nigeria Teaching Hospital Ituku Ozalla, Enugu State, Nigeria; 4 Department of Community Medicine, College of Health Sciences, Ebonyi State University Abakaliki, Ebonyi State, Nigeria; 5 African Institute for Health Policy and Health Systems, Ebonyi State University Abakaliki Ebonyi State, Ebonyi State, Nigeria

**Keywords:** HIV, Retention in care, Adherence to treatment, Hospital

## Abstract

**Background:**

Retaining patients on antiretroviral treatment in care is critical to sustaining the 90:90:90 vision. Nigeria has made some progress in placing HIV-positive patients on treatment. In an effort to increase access to treatment, ART decentralization has been implemented in the country. This is aimed at strengthening lower level health facilities to provide comprehensive antiretroviral treatment. We determined the level of retention and adherence to treatment as well as the associated factors among private and public secondary level hospitals in Anambra State.

**Method:**

We conducted a cross-sectional study among patients who had taken antiretroviral treatment for at least one complete year. A structured questionnaire and patient record review were used to extract information on patient adherence to treatment, and retention in care. Adherence to treatment was ascertained by patient self-report of missed pills in the 30 days prior to date of interview. Retention in care was ascertained using the 3-month visit constancy method reviewing the period spanning 12 months prior to the study.

**Result:**

We found a comparable level of retention in care (private 81.1%; public 80.3%; *p* = 0.722). However, treatment adherence was significantly higher amongst participants in the private hospitals compared to those in the public hospitals (private: 95.3%; public: 90.7%; *p* = 0.001). Determinants of good retention in the private hospitals included disclosure of one’s HIV status (AOR: 1.94, 95% CI: 1.09–3.46), being on first-line regimen (AOR: 3.07, 95% CI: 1.27–7.41), whereas being on once-daily regimen (AOR: 0.58, 95% CI: 0.36–0.92), and being currently married (AOR: 0.54 95% CI: 0.32–0.91) determined poor retention. In the public hospitals, only disclosure (AOR: 3.12 95% CI: 1.81–5.56) determined good retention, whereas, spending less than N1000 on transport (AOR: 0.230 95% CI: 0.07–0.78) and residing in a rural area (AOR: 0.64 95% CI: 0.41–0.99) determined poor retention. None of the factors determined adherence.

**Conclusion:**

Retention in care was high and comparable among the different hospital types and HIV disclosure status was an important factor relating to retention in care. The other factors that determined retention were however different at public and private hospitals. The HIV program manager should consider these variations in designing programs to improve patient retention in care and adherence to treatment.

## Background

Provision of antiretroviral treatment to HIV patients has led to longer survival, better quality of life and reduction of HIV transmission. Adherence to treatment and retention in care are critical to achieving these outcome through viral suppression [1–5]. Effective care of people living with HIV/AIDS (PLWHA) requires that patients are provided with satisfactory care, adhere to their treatment regimen and are retained in care. Adherence to therapy is one of the primary determinants of treatment outcome. Some of the major objectives of care and treatment of HIV/AIDS are to reduce morbidity and mortality as well as improve the quality of life of the PLWHA. These objectives among other factors are intricately linked to the achievement of optimal viral suppression which in turn depends on the level of adherence of the patient to treatment. Sustained period of good adherence (not less than 95%) to therapy is required to achieve adequate viral suppression which in turn is a predictor of good clinical outcome.

Poor adherence on the other hand is not only associated with poorer clinical outcome but also with a risk of developing drug resistance. Poor adherence reduces the optimal clinical outcome and the overall effectiveness of the treatment goal or target. The factors directly affecting patients’ level of adherence are diverse. They could be patient-related, treatment-related among others. Complexity of regimen (dosing frequency, pill burden), treatment side effects, poor health literacy, and poor patient-physician relationship have been reported to affect adherence to treatment [2].

Retention in care has variously been defined as “a spectrum of continuum of care packages starting from diagnosis of HIV infection till lifelong services” [6], “being alive and on ART or being transferred out to other health facilities to continue treatment” [7], and “as patients known to be alive and receiving highly active ART at the end of a follow up period” [8]. There is no gold standard in measuring retention in care [9, 10]. However, for a PLWHA to achieve viral suppression, he or she needs to be retained in care. Systematic reviews of retention in care in sub-Saharan Africa have shown that there was an increase in the level of estimated retention in care after two years on treatment from about 62% to about 76% over a period of three years (2007–2010) [8, 11].

Following decentralization of treatment from tertiary hospitals to secondary level hospitals in Nigeria, private hospitals have become intricately involved in the provision of such services due to their peculiar client base. Yet, studies are scarce that compare the treatment outcome in public and private hospitals in order to guide the policy direction for further scale-up activities. To help fill this gap, this study was carried out to compare retention in care, and adherence to treatment among adult HIV patients already on antiretroviral treatment in public and private secondary level hospitals in Anambra State, Nigeria.

## Methods

We conducted a comparative cross-sectional study among adult patients receiving antiretroviral treatment in secondary level hospitals in Anambra State. The hospitals were selected from the list of hospitals providing HIV treatment in the state. The participants were recruited through a multistage probability sampling technique. The details of the study sites, sample size determination and the sampling technique had been discussed fully in an earlier publication [12].

We included 1270 patients who had been on antiretroviral drug treatment for at least one complete year prior to the time of the study, had not been hospitalised or incarcerated for more than one month in the last one year prior to the study and were at least 18 years of age. ‘Transferred-in’ patients (patients who commenced their antiretroviral treatment in another hospital but who transferred to the index hospital to continue their treatment) were included in the study if they had completed at least one year of treatment in their current hospital before the beginning of the study. Patients whose complete medical records could not be seen (those using temporary case note with limited information) and those who were currently admitted in the hospital were excluded from the study.

### Study instrument

We administered a structured pretested questionnaire on consenting participants. The questionnaire captured information about the participants’ socio-demographic and clinical characteristics, as well as the factors affecting retention in HIV care and adherence to antiretroviral treatment. We extracted the patients’ adherence record and clinic visits from the patient’s case note.

### Measurement of variables

We assessed retention in care using the 3-month visit constancy method [9, 13, 14]. Three months visit constancy method was used because the appointment scheduling team in the study area schedules refill appointments every two months for stable patients and shorter for sick or non-adherent patients. The three months method is more sensitive compared to four-month method which is usually used in context where refill visits is scheduled every three months. Patients’ visit records for 12 months immediately prior to the study were reviewed. The year was divided into four equal quarters (three months in each quarter). Participants who visited the hospital and received antiretroviral refill at least once in each quarter for all the four quarters were classified as having good retention. Otherwise, they were classified as having poor retention.

We assessed adherence to ART using patient self-report of missed dose in the last one month. We then calculated the proportion of respondents with good adherence to ART based on the expected number of doses the patient had taken in the last 30 days prior to the interview. Participants who took ≥95% of the expected doses of their ART in the preceding 30 days were classified as having good adherence while those that took below 95% of their ART were classified as having poor adherence. The details of the calculation have earlier been documented [12].

The other explanatory variables assessed were socio-demographic characteristics (age, sex, education, place of residence, marital status, employment status) clinical characteristics (regimen type, regimen dosing frequency), HIV disclosure status and transportation cost.

### Data analysis

The data were entered and analyzed using Epi Info 7.0. We calculated the frequency and proportion of the participants who had good retention and good adherence. The proportion of patients who had good retention and those who had good adherence were compared across public and private hospitals using a Chi-squared test. The relationship between good retention in care and socio-demographic / clinical characteristics was assessed using Chi-squared test. Similarly, the relationship between good adherence to ART and socio-demographic / clinical characteristics was explored using Chi-squared test. We used a *p* value of 0.1 as a cut-off in selecting variables in the bivariate analysis to be used in the logistic regression model. The variables were examined for collinearity and variance inflation factor less than 3 was set as cut-off. We estimated the adjusted odds ratio, the 95% confidence interval and a *p* value was calculated. Significance was then determined from a *p* value < 0.05.

## Results

A total of 1,234 (97.2%) participants were on first-line regimen (public: 98.0%; private: 96.4%). The participants had spent a comparable period on treatment since commencing of antiretroviral treatment (mean months on treatment for public participants: 53.3 ± 30.0; private: 50.1 ± 27.5). The difference between them was not statistically significant (*p* = 0.160). Participants in public hospitals were more likely to be on 24-hourly medications compared to those in private hospitals (public: 28.2%; private: 21.7%; *p* = 0.008). The pill burden was however comparable in the two groups of patients (p = 0.09).

More participants in the public hospitals reported missing at least a dose of medication in the preceding four weeks (33.4%) when compared to private hospital participants (17.2%): the difference being statistically significant (*p* < 0.001; Table 1). The major reasons given by the participants for the missed doses were similar in both public and private health facilities. The reasons included forgetfulness (public: 44.8%, private: 42.2%), being away from home (public: 25.9%, private: 36.7%), running out of medicines (public: 6.6%, private: 11.0%), difficulty taking the medication at the specified time (public: 5.7%, private: 7.3%) and drug side effect (public: 3.8%, private: 3.7%). Public health facility participants reported lower proportion of good ratings on their ability to take the medication as prescribed (94.8%) when compared to the participants in the private health facility (96.7%): the observed difference in proportion being however not statistically significant (*p* = 0.095).

**Table 1:** Participants’ reporting missed doses and reasons for missed doses

Variable	Public (N = 635) frequency (%)	Private (*N* = 635) frequency (%)	Chi square (χ^2^)	*p*-value
Missed at least one dose of HIV drug in the past 4 weeks				
Yes	212 (33.4)	109 (17.2)	44.23	< 0.001
	*n* = 212	*n* = 109		
Reasons for missed doses by the participants (*n* = 321)				
Forgetfulness	95 (44.8)	46 (42.2)		
Away from home	55 (25.9)	40 (36.7)		
Ran out of drugs	14 (6.6)	12 (11.0)		
Inconvenient timing	12 (5.7)	8 (7.3)		
Drug side effect	8 (3.8)	4 (3.7)		
Too many drugs to take	7 (3.3)	1 (0.9)		
Confusion on how to take the drugs	4 (1.9)	3 (2.8)		
Fasting	4 (1.9)	0 (0)		
Lack of privacy	4 (1.9)	0 (0)		
Others	7 (3.3)	8 (7.3)		
Participants rating of their ability to take medication as prescribed				
Good^b^	602 (94.8)	614 (96.7)	2.79	0.095
Poor^a^	33 (5.2)	21 (3.3)		

^a^Included The Ratings Fair, Poor, Very Poor

^b^Included the ratings good, very good, excellent

The HIV status disclosure rate by participants was comparable between the public (89.1%) and the private (88.3%) hospitals (*p* = 0.657; Table 2). The disclosures were mainly to spouse, siblings and parents (close family relatives). Participants accessing their care in private health facilities were more likely to disclose to their spouses (64.5%, p < 0.001), while the participants accessing their treatment in public health facilities were more likely to disclose to their siblings (43.8%, *p* < 0.001) and others (13.4%). The other disclosure rates were comparable in the two groups.

**Table 2:** Participants’ disclosure of HIV status

Variable	Public (N = 635) frequency (%)	Private (N = 635) frequency (%)	Total (*N* = 1270) frequency (%)	Chi square (χ^2^)	*p*-value
Participants who have disclosed their HIV status to someone					
Yes	566 (89.1)	561 (88.3)	1127 (88.7)	0.18	0.657
No	69 (10.9)	74 (11.7)	143 (11.3)		
Who the Participants disclosed their HIV status to^a^	*N* = 566	*N* = 561	*N* = 1127		
Spouse	301 (53.2)	362 (64.5)	663 (58.8)	11.74	< 0.001
Siblings	248 (43.8)	170 (30.3)	418 (37.1)	21.70	< 0.001
Parents	107 (18.9)	104 (18.5)	211 (18.7)	0.05	0.821
Friends	31 (5.5)	28 (5.0)	59 (5.2)	0.16	0.689
In law	7 (1.2)	8 (1.4)	15 (1.3)	0.07	0.795
Colleague at work	5 (0.9)	6 (1.1)	11 (1.0)	0.09	0.762
Neighbour	7 (87.5)	1 (12.5)	8 (0.6)	3.15	0.076
Others^b^	76 (13.4)	45 (8.0)	121 (10.7)	8.78	0.003

^a^Multiple response allowed

^b^Children

The proportion of participants who had good retention in care was comparable in both private (81.1%) and public (80.3%) hospitals (*p* = 0.722). However, greater proportion of participants in the private hospitals (95.3%) had good adherence to ART compare to those in the public (90.7%) hospitals (*p* = 0.001; Table 3).

**Table 3:** Retention in care and adherence to treatment among HIV patients receiving treatment from secondary level hospitals in Anambra State, Nigeria

Variable	Public (*n* = 635) Frequency (%)	Private (*n* = 635) Frequency (%)	Chi square (χ^2^)	*p*-value
Retention in care				
Adequate retention	510 (80.3)	515 (81.1)	0.126	0.722
Inadequate retention	125 (19.7)	120 (18.9)		
Adherence to treatment				
Adequate adherence	576 (90.7)	605 (95.3)	10.16	0.001
Inadequate adherence	59 (9.3)	30 (4.7)		

Table 4 shows the relationship between participants’ socio-demographic characteristics and adequate retention in care in each of the hospital types. The factors with p value less than 0.1 in the bivariate analysis were modelled for each hospital in a logistic regression (Table 5).

**Table 4:** Relationship between socio-demographic/clinical characteristics and retention in care in public and private hospitals in Anambra State, Nigeria

Variable	Public hospital			Private hospital		
	Good retention (%)	COR (95% CI)	*p*-value	Good retention (%)	COR (95% CI)	*p*-value
Age						
Less than 35 yrs	135 (75.7)	0.7 (0.44–1.00)	0.048	164 (82.4)	1.13 (0.73–1.75)	0.569
35 yrs. and older	357 (82.5)			351 (80.5)		
Gender						
Male	140 (82.4)	0.8 (0.53–1.31)	0.435	149 (78.8)	1.2 (0.80–1.88)	0.342
Female	370 (79.6)			366 (82.1)		
Marital Status						
Currently Married	300 (80.9)	1.1 (0.73–1.61)	0.681	381 (79.4)	0.6 (0.36–1.00)	0.050
Not Currently married^a^	210 (79.6)			134 (134)		
Employment status						
Employed	451 (82.0)	2.0 (1.21–3.34)	0.007	465 (81.3)	1.1 (0.59–2.15)	0.711
Unemployed	59 (69.4)			50 (79.4)		
Place of residence						
Rural	154 (76.2)	0.7 (0.46–1.04)	0.078	165 (84.6)	1.4 (0.90–2.22)	0.132
Urban	356 (82.2)			350 (79.6)		
Education						
Primary education or less	224 (84.5)	1.6 (1.06–2.42)	0.024	107 (74.8)	0.6 (0.39–0.95)	0.029
Secondary education and more	286 (77.3)			408 (82.9)		
Dosing frequency						
12 hrly	371 (81.4)	0.8 (0.52–1.22)	0.291	412 (82.9)	0.6 (0.39–0.95)	0.028
24 hrly	139 (77.7)			103 (74.6)		
Regimen type						
First line	502 (80.7)	2.6 (0.84–8.13)	0.085	501 (81.9)	2.9 (1.22–6.87)	0.012
Second line	8 (61.5)			14 (60.9)		
Experienced stock out						
Yes	256 (85.1)	1.8 (1.2–2.68)	0.004	20 (74.1)	0.7 (0.27–1.58)	0.340
No	254 (76.1)			495 (81.4)		
Disclosure status						
Yes	471 (83.2)	3.8 (2.26–6.44)	< 0.001	461 (82.2)	1.7 (0.98–2.98)	0.057
No	39 (56.5)			54 (73.0)		
Transportation cost						
Less than 1000	470 (79.4)	0.3 (0.09–0.95)	0.030	455 (80.7)	0.8 (0.39–1.50)	0.437
More than 1000	40 (93.0)			60 (84.5)		

^a^ = Not currently married included Single (never married), Separated, Divorced and Widowed COR = Crude odds ratio

**Table 5:** Multivariable logistic regression of factors associated with adequate patients’ retention in public and private hospitals

Independent variable	Public hospital			Private hospital		
	Adjusted Odds Ratio	(95% CI)	*p*-value	Adjusted Odds Ratio	(95% CI)	*p*-value
Age						
Less than 35	0.9	0.56–1.37	0.566			
35 years and more	1					
Education						
Primary education or less	1.4	0.90–2.19	0.135	0.7	0.42–1.05	0.077
Secondary education or more	1			1		
Marital status						
Currently married				0.5	0.32–0.91	0.021
Not currently married				1		
Employment status						
Employed	1.6	0.90–2.74	0.112			
Unemployed	1					
Place of residence						
Rural	0.6	0.41–0.99	0.045			
Urban	1					
Dosing frequency						
24 hourly				0.58	0.36–0.92	0.021
12 hourly				1		
Regimen type						
First line	2.5	0.79–8.18	0.120	3.1	1.27–7.41	0.013
Second line	1			1		
Experienced stock out						
Yes	1.7	1.09–2.54	0.018			
No	1					
Disclosure status						
Yes	3.2	1.81–5.56	< 0.001	1.9	1.09–3.46	0.024
No	1			1		
Transportation Cost (N)						
1000 and below	0.2	0.07–0.78	0.019			
More than 1000	1					

Marital status, regimen type, ART dosing frequency and disclosure status were statistically significant predictors of retention among participants accessing care in private health facilities (Table 5). Participants who had disclosed their status had 1.94 odds of being retained in care compared to those who had not (*p* = 0.024). Similarly, participants who were on first-line regimen had 3.067 odds of being retained compared to those on second-line regimen (*p* = 0.013).

Table 6 shows the relationship between the socio-demographic and clinical characteristics of the participants, in relation to their adherence to antiretroviral treatment. The factors with *p* value < 0.1 in the bivariate analysis were modelled in logistic regression (Table 7).

**Table 6:** Relationship between socio-demographic/clinical characteristics and patients’ adherence to treatment in public and private hospital

Variable	Public Hospital			Private Hospital		
	Good Adherence (%)	COR (95% CI)	*p*-value	Good Adherence (%)	COR (95% CI)	*p*-value
Age						
< 35 yrs	175 (86.6)	0.5 (0.30–0.89)	0.016	192 (96.5)	1.5 (0.64–3.62)	0.333
≥ 35 yrs.	401 (92.6)			413 (94.7)		
Gender						
Male	159 (93.5)	1.7 (0.84–3.28)	0.139	426 (95.5)	1.2 (0.55–2.59)	0.661
Female	417 (89.7)			179 (94.7)		
Marital Status						
Currently Married	342 (92.2)	1.5 (0.88–2.59)	0.129	457 (95.2)	0.9 (0.40–2.23)	0.889
Not Currently married	234 (88.6)			148 (95.5)		
Employment Status						
Employed	503 (91.5)	1.8 (0.89–3.47)	0.099	546 (95.5)	1.4 (0.48–4.22)	0.522
Unemployed	73 (85.9)			59 (93.7)		
Place of Residence						
Rural	182 (90.1)	0.9 (0.51–1.59)	0.718	188 (96.4)	1.5 (0.62–3.51)	0.370
Urban	394 (91.0)			417 (94.8)		
Education						
≤ Primary Education	248 (93.6)	1.9 (1.04–3.36)	0.035	136 (95.1)	1.0 (0.40–2.27)	0.913
≥ Secondary Education	328 (88.7)			469 (95.3)		
Dosing Frequency						
12hrly	417 (91.5)	0.7 (0.42–1.31)	0.306	473 (95.2)	1.1 (0.44–2.79)	0.814
24hrly	159 (88.8)			132 (95.7)		
Regimen Type						
First line	565 (90.8)	1.8 (0.39–8.33)	0.444	585 (95.6)	3.3 (0.91–11.61)	0.055
Second line	11 (84.6)			20 (87.0)		
Experienced Stock out						
Yes	270 (89.7)	0.8 (0.47–1.36)	0.406	22 (81.5)	0.2 (0.06–0.54)	< 0.001
No	306 (91.6)			583 (95.9)		
Disclosure Status						
Yes	514 (90.8)	1.1 (0.49–2.56)	0.796	533 (95.0)	0.5 (0.13–2.27)	0.383
No	62 (89.9)			72 (97.3)		

*COR* = Crude odds ratio

**Table 7:** Multivariable logistic regression of factors associated with patients’ adherence to treatment in public and private hospitals in Anambra State

Independent variables n = 635	Public hospital			Private hospital		
	Adjusted Odds Ratio	(95% CI)	*p*-value	Adjusted Odds Ratio	(95% CI)	*p*-value
Age						
< 35 years	0.6	0.34–1.05	0.074			
≥ 35 years	1					
Experienced Stock out						
Yes				0.2	0.07–0.60	0.004
No				1		
Regimen Type						
First line				2.7	0.71–10.00	0.145
Second line				1		
Education						
≤ Primary education	1.6	0.89–2.99	0.110			
≥ Secondary education	1					
Employment						
Employed	1.5	0.73–2.94	0.288			
Unemployed	1					

None of the factors modelled significantly predicted patient adherence to treatment among participants accessing care in public health facilities. However, among the private hospital participants, experience of stock-out of antiretroviral drugs was a significant negative predictor of adherence to treatment. Those that had experienced stock-outs had 0.206 odds of being adherent to their treatment compared to those who had not (*p* = 0.004).

## Discussion

The proportion of patients retained in care in both facility types was high. The high rate was commendable bearing in mind that it was a composite measure of retention for patients who had completed at least one year on antiretroviral therapy. The retention rates achieved among the participants were comparable with those found in other studies carried out in Nigeria, other parts of Africa and elsewhere [15–20]. Retention in care has been argued as a marker of quality of care and an important requirement for patients to achieve viral suppression and other important beneficial outcome in HIV treatment. A lower rate of retention was previously reported in a public tertiary hospital in the region [17]. It has been argued that, congestion in the tertiary hospitals and occasional excessive demand on their scarce resources, makes it difficult for the tertiary hospitals to retain most of their patients.

The high rate of retention has a great significance for the decentralization efforts of the government in making ART services more accessible to the populace. There have been fears that lower level health facilities do not have the capacity to provide comprehensive treatment for people living with HIV. Despite these fears, there was a need to decentralize care to more centres since the public tertiary hospitals were few and far apart. This made the provision of these services difficult as patients had to travel long distances and spend more hours trying to access care, with the consequent effect on quality of services and retention. The high rates observed in these types of hospital support the fact that secondary health facilities could effectively provide these treatments. The comparability of the rates in both facility types signifies that private health facilities had the capacity to actively engage and retain a substantial number of their clients on lifelong therapy in care. These findings support the addition of private facilities that meet the criteria to provide comprehensive HIV care and treatment. This would help increase geographical access to treatment for a greater number of patients and reduce their transportation costs.

This study revealed a high level of patient adherence to treatment in both public and private health facilities. It is noteworthy that all the health facilities that participated in the study had a functional adherence unit. The units were involved in treatment preparation of patients before they were commenced on ART and provided ongoing adherence counselling and support to all those on treatment. The high levels of adherence observed in this study was comparable to the other studies in Nigeria and Africa [21–25]. The proportion of patients with good adherence to treatment was however higher than that reported in two other studies in Togo and Nepal which reported adherence levels of 78.4% [26] and 85% respectively [27]. In the study that reported an adherence level of 78.4%, a composite measure combining the patient self-report, pill count and appointment adherence was used to calculate the global composite adherence as against the patient self-report used in our study and that could have resulted in the lower value. Similarly, the study that reported 85% level of adherence used total adherence (that is 100% adherence with no missed dose during the period of observation) instead of the 95% standard level which could have accounted for the recording of a lower adherence level.

The difference in adherence between the two hospitals, though small, might become clinically important especially when the numbers are large. The most feared consequence of poor adherence to ART is the development of resistance to ART. The resistance developed is transmissible to other individuals who become resistance even before being exposed to ART. The implication of this in an environment with HIV epidemic needs immediate intervention to address.

The proportion of participants who had missed at least a dose of their medication was significantly higher in public health facilities. The higher rate of missed doses among participants in public health facilities, which was twice as high as observed in the private health facilities, is a cause for concern. The high proportion of participants who had missed at least one dose of their ART in the preceding four weeks could have explained the difference observed in the adherence rate in public and private health facility. This calls for an effort to intervene in the factors militating against the participants’ ability to adhere completely to their medications. The finding is in keeping with that reported earlier in Anambra [22] but lower than the rate reported in Uganda [21]. The higher proportion of participants who had missed at least one dose of their medication observed in the Ugandan study was probably because of the longer duration of period under review (“ever fail to take ones ARV drugs”) as against one-month review period used in this study.

The main reasons adduced for the missed doses were forgetfulness, being away from home, running out of drugs, difficulty taking drugs at specified time, and drug side effects. The reasons observed in this study was in keeping with those observed in similar studies done in Nigeria and Africa [21–24, 28, 29]. Forgetfulness and being away from home (or drug) at the time the medication should be taken were the commonest reasons for missing medication. Efforts targeted at reminding the patients at the time that they should take their medication and having their drugs with them while away from home could help improve the level of adherence among the participants. The use of alarms, pillboxes, text messages and calendars have been reported to assist patients improve their adherence [30–32]. Efforts aimed at personalizing adherence counselling and tying the time of medication intake to patient routines could also help improve adherence levels.

Participants in public health facilities also reported higher frequencies of stock-out (inability to receive their antiretroviral drugs on scheduled visit date due to non-availability at the health facility) in the twelve months preceding the study. This may have contributed to the poor adherence level, as running out of drugs were among the factors given by patients for missing their medications. Patients that were not able to get their drugs as scheduled were more at risk of running out of their medication. The public health facilities need to devise better strategies of managing their drug supply chain to minimize incidences of antiretroviral drug stock out in the facilities.

The level of adherence was significantly higher in private compared to the public health facilities. This could have been due to multi-dimensional nature of the adherence to treatment concept which goes beyond the health worker patient interaction to other patient-centred and treatment-centred factors. It could probably be due to more stringent and personal measures taken by the adherence units of the private hospitals studied. One of the practices observed was more frequent and personalized follow-up calls and visits especially when patients missed their appointments. They were done usually within a day or two after missing a scheduled appointment. This finding highlights the need, in the public hospitals, for a more personalized follow-up of patients on treatment especially when they miss their scheduled appointments soon after the appointments. Such strategies have similarly been reported to promote retention in private facilities in the study area [17]. The follow up calls were done usually within a day or two after missing a scheduled appointment. This finding further justifies the need for more personalized and timely follow up of patients on treatment especially soon after they miss their scheduled appointments.

The variation in the predictors between public and private health facilities could be due to the differences observed in the socio-demographic characteristics. The effect of disclosure of one’s HIV status to retention in care could be due to the social support it provides. It has been reported that non-disclosure of HIV status was associated with poor retention in care [33]. Efforts at encouraging patients to disclose their status to close family relatives and friends which was observed as a common practice among the study population, should be encouraged. Other benefits associated with disclosure of HIV status have been reported [34].

Among the participants in the public hospitals, none of the factors modelled significantly predicted patient’s adherence to treatment. However, among participants in the private hospitals, experience of stock-out was a negative predictor of adherence to treatment among the private health facilities’ participants. A multicentre study involving five tertiary public health facilities in Nigeria concluded that HIV disclosure status to either spouse or family member and Tenofovir-containing first-line regimens were significant positive predictor of good adherence [35]. Other studies have also reported different predictors of adherence to treatment in different populations and settings. Disclosure of HIV status [36, 37], education [36], duration on ART treatment [37] and alcohol use [36] have all been reported as predictors of adherence to treatment. These were not however elicited in this study.

Our study assessed the retention in care among those who have already spent at least one year on antiretroviral treatment. This may limit the generalization of our study. We were not able to assess retention among patients within the first year of beginning ART neither did we assess long term longitudinal retention. Combining all the patients who have been on ART for at least one year might have masked the variations that could have existed over the different periods on treatment. There is need for qualitative study to understand the underlying reasons for the differences observed in the two hospital types.

## Conclusion

Our study further strengthens the findings that support decentralization of HIV services to lower secondary level hospitals. It also reveals that secondary level hospitals were able to retain HIV patients while recording high level of adherence to antiretroviral among the patients. The factors that determine who is retained were however different in  public and private hospitals. The HIV program manager are therefore encouraged to consider these variations in designing programs aimed at improving patient retention and adherence to treatment.

## Data Availability

The data generated in the course of the study is freely available online at (10.3886/E103460V1).

## Abbreviation

AIDS: Acquired Immunodeficiency Syndrome
AOR: Adjusted odds ratio
ART: Antiretroviral treatment
ARV: Antiretroviral
CI: Confidence interval
HIV: Human immunodeficiency virus
PLWH: People living with HIV/AIDS
